# Case Report: Effect of a graded task-oriented throwing training on throwing accuracy and kinematic variability in a baseball player with the yips

**DOI:** 10.3389/fspor.2025.1636650

**Published:** 2025-08-29

**Authors:** Toshiyuki Aoyama, Rinri Uematsu, Satoshi Shibata, Kazumichi Ae, Takashi Kawamura, Yutaka Kohno

**Affiliations:** ^1^ Department of Physical Therapy, Ibaraki Prefectural University of Health Sciences, Ami-Machi, Ibaraki, Japan; ^2^ Graduate School of Comprehensive Human Sciences, University of Tsukuba, Tukuba, Ibaraki, Japan; ^3^ Faculty of Sport Culture, Nippon Sport Science University, Setagaya, Japan; ^4^ Institute of Health and Sport Sciences, University of Tsukuba, Tukuba, Ibaraki, Japan; ^5^ Centre for Medical Sciences, Ibaraki Prefectural University of Health Sciences, Ami-Machi, Ibaraki, Japan

**Keywords:** yips, baseball, kinematics, task-oriented training, variability

## Abstract

Despite the high prevalence of the yips, a task-specific movement disorder in athletes, effective exercise therapy remains elusive. This case report aimed to evaluate the impact of task-oriented training on throwing accuracy and kinematic variability in a baseball player with the yips. This study included a 21-year-old baseball player with a 7-year history of the yips. Approximately 50% of the player's typical throws were erratic and uncatchable. However, the frequency of erratic throwing varied depending on the throwing task and its contexts. To address these symptoms, graded task-oriented throwing training was implemented twice a week for 5 weeks. A three-dimensional motion capture system and high-speed camera were employed to assess throwing accuracy and kinematic variability. After the intervention, the frequency of subjective yips symptoms during throwing decreased by approximately one-third. Moreover, the intervention improved the accuracy of the ball arrival position and reduced variability in the ball release angle and shoulder internal rotation angle at ball release. Treating the yips remains challenging owing to concerns regarding doping and side effects. Nonetheless, this study suggests that low-risk physical therapy may have potential clinical utility as a management approach for athletes with the yips.

## 1 Introduction

The yips is a psychoneuromuscular disorder featuring involuntary movements that prevent the execution of coordinated sports movements (1–3). Classically, the yips can be classified into two subtypes, namely task-specific dystonia (Type I) and choking (Type II) (4); the former involves physical symptom complaints, whereas the latter pertains to psychological symptoms. Smith et al. reported a 28% incidence of the yips among golfers (1). Additionally, 47% of college baseball players experienced yips-related symptoms (5). Considering the high prevalence and substantial impact of the yips on athletes' careers, effective intervention strategies must be developed. Some studies examining the effects of psychological treatment for the yips have reported a reduction in their symptoms (6–8). Alternatively, yips symptoms can also appear without being associated with psychological factors. Therefore, intervention strategies other than psychotherapy would be required for nonpsychological symptoms. Medical treatments, such as drug therapy, injection therapy, and surgery, could be potential strategies for the treatment of these symptoms (9, 10). However, side effects and doping issues must be considered when applying medical treatments to athletes. Furthermore, studies have shown that symptoms of the yips were task- or context-dependent (5, 11). For example, the symptoms among baseball players can fluctuate considerably depending on factors other than those psychological in nature, such as throwing distance and throwing strength (5, 11). With this background, we believe that graded task-oriented training (12, 13), which modifies motor tasks or their contexts based on the characteristics of yips symptoms, may be a useful intervention, especially for athletes with nonpsychological yips symptoms. To date, no study has yet clarified whether task-oriented training is effective in treating the yips symptoms. We herein present a case in which throwing training using a task-oriented approach decreased throwing error and kinematic variability in the throwing motion in a baseball player with the yips.

## 2 Methods

### 2.1 Case presentation

Our case involved a 21-year-old male right-handed baseball player who started playing baseball at the age of 9. Although he had no orthopedic or neurological issues, he began experiencing throwing yips at age 14. The onset was triggered by a game-losing throwing error. After that event, he frequently made erratic throws—often so inaccurate that his partner could not catch them—even in low-pressure situations without any psychological stress. He retired from playing baseball at the competitive level due to symptoms of the yips 3 years prior to his participation in this study, but continued to play it at the recreational level. The symptoms continued until his participation in this study.

The player satisfied the criteria for throwing yips (3, 11). He demonstrated no muscle weakness or restricted range of motion that would impair his ability to throw. A neurological evaluation confirmed the absence of abnormalities such as motor paralysis or involuntary movements. However, he reported a noticeable sense of excessive muscle tension in his arms. These characteristics are consistent with task-specific dystonia (4).

To explore psychological contributors, personality traits frequently linked to yips—neuroticism and agreeableness—were assessed using the NEO Five-Factor Inventory (11), yielding scores of 27 and 28, respectively. Furthermore, his anxiety levels were measured with the State-Trait Anxiety Inventory, with scores of 27 and 42 for state and trait anxieties, respectively. All scores were below the national average for his age and sex (14, 15). Although the yips were triggered by a psychologically stressful event, his psychological profile was not a primary factor in the onset or persistence of his symptoms.

During standard throws over a distance of approximately 10 m, nearly half of his attempts were inaccurate. However, the intensity of his symptoms substantially varied depending on the specific throwing task and context. Our assessment revealed several factors affecting his throwing accuracy, including posture, leg movement patterns, arm cocking motion, and the type of ball used. Notably, his symptoms did not appear while throwing from a seated position, even before the intervention. Furthermore, his symptoms were less severe when executing a “short-arm” throw—characterized by a reduced arm extension during the cocking phase—compared with his standard throwing technique. In addition, throws incorporating a forward stepping motion (shuffle throws) resulted in fewer errant throws, defined as throws that were very difficult for the receiving partner to catch due to poor accuracy, than his usual form. The size of the ball had a noticeable effect: larger balls, such as those used in handball or softball, were less likely to trigger his symptoms. In contrast, variations in throwing effort or distance did not considerably impact the severity of his symptoms (5, 11). The player provided written informed consent to participate in this study. All experiments were performed in accordance with the Declaration of Helsinki, and the study protocols were approved by the ethics committee of the university, to which the first author belongs (Approval no. 926).

### 2.2 Throwing task

Before measuring the throwing motion, the player performed his own full warm-up routine. He was instructed to throw a ball as forcefully as possible toward a circular target (200 mm in diameter) located 10 m away. At least 10 trials were performed. After each throw, he reported whether or not his symptoms of the yips were reproduced.

### 2.3 Measurement of throwing accuracy

The ball arrival position was filmed using a high-speed camera (Sony, DSC-RX100M5A; frame rate, 240 fps; shutter speed, 500/s), and measured using scientific image analysis software (Image J). Regarding the center coordinates of the target, positive values were defined as upward and rightward in the vertical and horizontal directions, respectively. The vertical and horizontal error distances were measured, from which the total error was calculated.

### 2.4 Motion capture system

A gold-standard three-dimensional motion capture system (VICON, UK; 250 Hz) was used to analyze the throwing motion (16). Reflective markers were placed on 30 anatomical landmarks, including the index fingernail, head of the third metacarpal, radial and ulnar styloid processes, medial and lateral humeral epicondyles, anterior and posterior shoulder joints, acromion of the throwing arm, head vertex, C7, T10, xiphoid process, bilateral tragions, 10th ribs, medial and lateral knees, heels, toes, and medial and lateral malleoli. Furthermore, six reflective markers were affixed to the ball. For each throwing motion, the duration from the posterior point (nonthrowing direction) of the ball's trajectory to ball release was normalized to 101 data points (0%–100% of the throwing cycle) using cubic spline interpolation.

**Figure 1:**
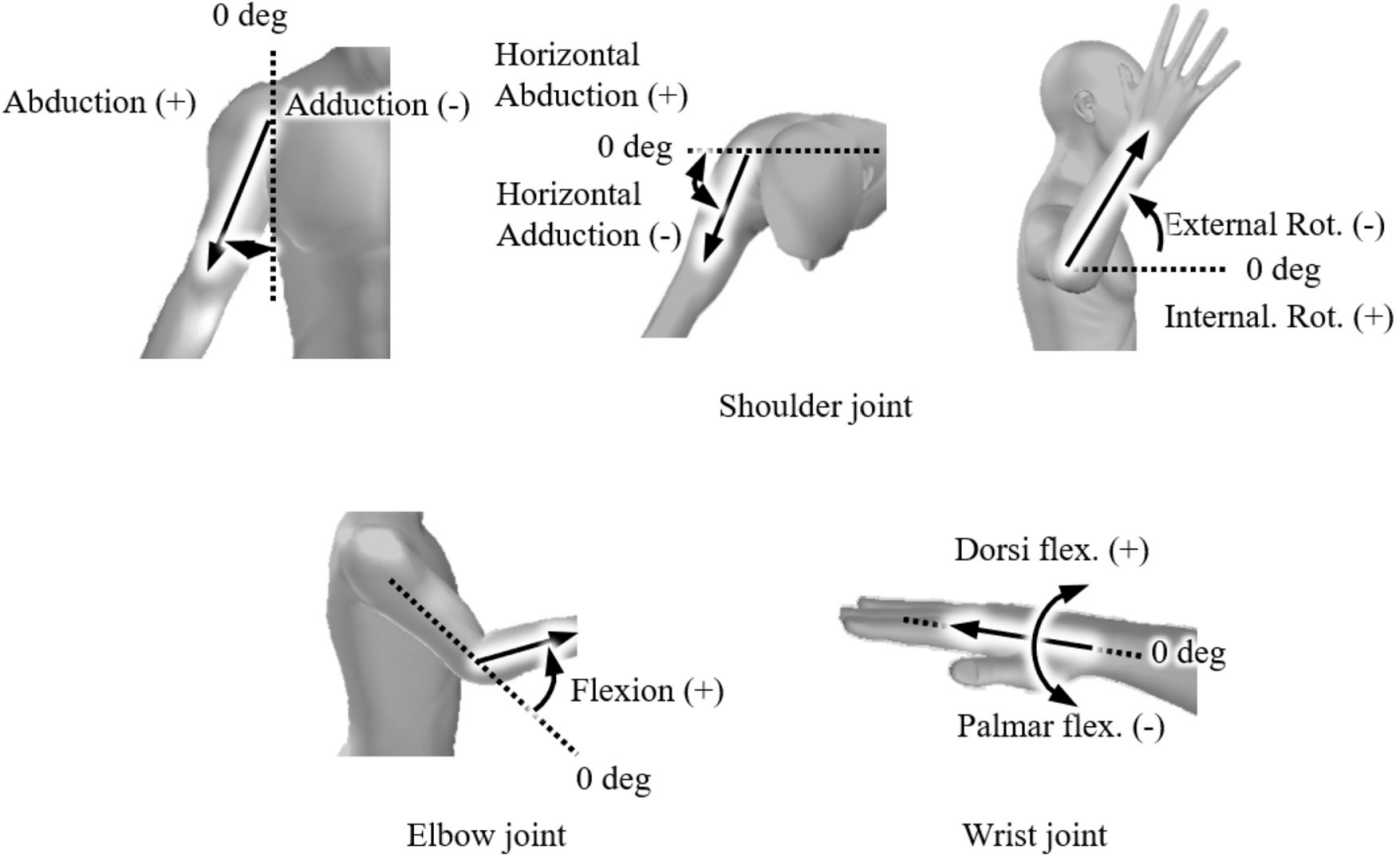
Definition of the upper limb joint angles.

A world coordinate system was defined using the *X* axis as the throwing direction, the Z axis as the vertical upward direction, and the *Y* axis as the direction obtained by the cross product of the X and Z axes. The tip of the pivot foot before the throwing motion was defined as the origin of the coordinate system. The vertical ball release angle in the X–Z plane and the horizontal ball release angle in the X–Y plane were then calculated, with positive values for the upward and rightward (third base direction) directions. Figure 1 illustrates the definitions of each joint angle. The Euler angles of the right upper arm relative to the upper torso defined the shoulder joint angles. Shoulder joint movements were defined as abduction/adduction, horizontal abduction/adduction and externa/internal rotation angles in the order of rotation, with positive values indicating the direction of abduction, horizontal abduction and internal rotation, respectively. The Euler angle of the right forearm coordinate system relative to the right upper arm coordinate system was defined as the right elbow joint angle. The elbow flexion/extension angle was calculated, with flexion defined as a positive value. The wrist joint angle was determined using the Euler angle of the right hand coordinate system relative to the right forearm. For the wrist, dorsiflexion was assigned a positive value.

### 2.5 Graded task-oriented throwing training

Considering the task- and context-dependent characteristics of yips symptoms described earlier, we developed a graded, task-oriented throwing training program. Task-oriented training emphasizes repetitive, context-specific movements that target the motor skills necessary for performing goal-directed functional tasks (12). This approach effectively rehabilitates individuals with stroke (12), Parkinson's disease (17), and cerebral palsy (18). Therefore, this study applied task-oriented training to address yips symptoms in a baseball player.

We designed a progressive throwing program customized to the athlete's unique symptom profile. The training began with low-demand tasks that did not provoke yips symptoms—such as throwing from a seated or kneeling position—allowing him to maintain greater control and focus on improving accuracy. Before the intervention, we identified several factors influencing symptom severity, including posture (sitting, kneeling, lunge, and standing), ball type (baseball, softball, and handball), leg movement (no step, left-leg stride, and forward step), and arm motion style (short- and long-arm). These variables were systematically adjusted over a 5-week period to gradually increase task complexity while minimizing performance breakdown. From Table 1, each training stage was designed to keep errant throws, which were objectively judged based on whether the receiving partner could reasonably catch the ball, to fewer than 1 or 2 out of 10 attempts. The athlete participated in supervised sessions two times weekly for 1 h, complemented by twice-weekly unsupervised sessions using less demanding tasks to reinforce skill retention and prevent maladaptive patterns. Each session typically involved five drills, with 10–30 throws per drill.

**Table 1:** Timeline of the training sequence.

Week	Posture	Ball type	Leg movement	Arm movement
1	Sitting	Baseball	No step	Short arm
1	Kneeling	Baseball	No step	Short arm
1	Standing	Handball	No step	Short arm
1	Standing	Handball	Stride with left leg	Short arm
2	Standing	Softball	No step	Short arm
2	Standing	Softball	Forward steps (Shuffle throw)	Short arm
2	lunge	Softball	No step	Short arm
2	Standing	Softball	Stride with left leg	Short arm
2	Standing	Baseball	No step	Short arm
3	Standing	Baseball	Forward steps (Shuffle throw)	Short arm
3	lunge	Baseball	No step	Short arm
3	Standing	Baseball	Stride with left leg	Short arm
3	Standing	Handball	No step	Long arm
4	Standing	Handball	Stride with left leg	Long arm
4	Standing	Softball	No step	Long arm
4	Standing	Softball	Forward steps (Shuffle throw)	Long arm
4	lunge	Softball	No step	Long arm
4	Standing	Baseball	No step	Long arm
5	Standing	Baseball	Forward steps (Shuffle throw)	Long arm
5	lunge	Baseball	No step	Long arm
5	Standing	Softball	Stride with left leg	Long arm
5	Standing	Baseball	Stride with left leg	Long arm

• Sitting/kneeling: throwing motion initiated from a seated or kneeling posture without stepping.

• Lunge: throwing motion performed from a lunge posture, with the lead leg positioned forward prior to the initiation of the throwing motion.

• No step: throwing motion executed without any forward stepping or lead leg movement, with both feet remaining stationary throughout the throw.

• Stride with left leg: throwing motion performed with a standard forward stride of the lead leg.

• Forward steps: throwing motion incorporating forward stepping movements taken prior to the initiation of the throwing motion.

• Short arm: throwing motion performed without full arm extension during the cocking phase.

• Long arm: throwing motion performed with full arm extension during the cocking phase.

## 3 Results

### 3.1 Subjective evaluation

The self-reported frequency of yips symptoms decreased from 80% preintervention to 27% postintervention.

### 3.2 Throwing accuracy

Table 2 summarizes the results of the throwing accuracy. After the intervention, variability (standard deviation) in ball arrival position decreased by 11.6% horizontally and 28.7% vertically. Error distances were also reduced—by 23.2% and 36.8% in the horizontal and vertical directions, respectively—leading to a 33.8% overall reduction in total error distance. The variability in ball release angle decreased by 18.5% horizontally and 25.6% vertically.

**Table 2:** Results for throwing accuracy and kinematic data.

Variables	Pre		Post		% change in variability
	Average	SD	Average	SD	
Ball arrival position (m)					
Horizontal ball arrival position	−0.199	0.448	0.072	0.396	−11.6
Vertical ball arrival position	−0.155	0.694	0.017	0.495	−28.7
Error distance (m)					
Horizontal error distance	0.422	0.230	0.324	0.217	−23.2
Vertical error distance	0.631	0.285	0.399	0.265	−36.8
Total error distance	0.795	0.273	0.526	0.322	−33.8
Ball release angle (degree)					
Horizontal ball release angle	−0.3	2.7	−4.3	2.2	−18.5
Vertical ball release angle	−0.6	3.9	1.8	2.9	−25.6
Ball release position (m)					
Horizontal ball release position	0.319	0.028	0.319	0.028	0
Antero-posterior ball release position	1.237	0.059	1.340	0.038	−35.6
Vertical ball release position	1.675	0.023	1.602	0.016	−30.4
Joint angle at ball release (degree)					
Shoulder abduction	82.3	1.7	82.3	1.1	−35.3
Shoulder horizontal abduction	−25.5	2.1	−28.0	0.7	−66.7
Shoulder internal rotation	48.8	9.2	52.7	5.9	−35.9
Elbow flexion	29.3	1.1	32.0	1.4	27.3
Wrist dorsiflexion	4.5	4.5	−4.7	6.1	35.6

Percentage change in variability was calculated from the average values for error distance and from the standard deviation values for all other measures.

### 3.3 Ball release parameters

**Figure 2:**
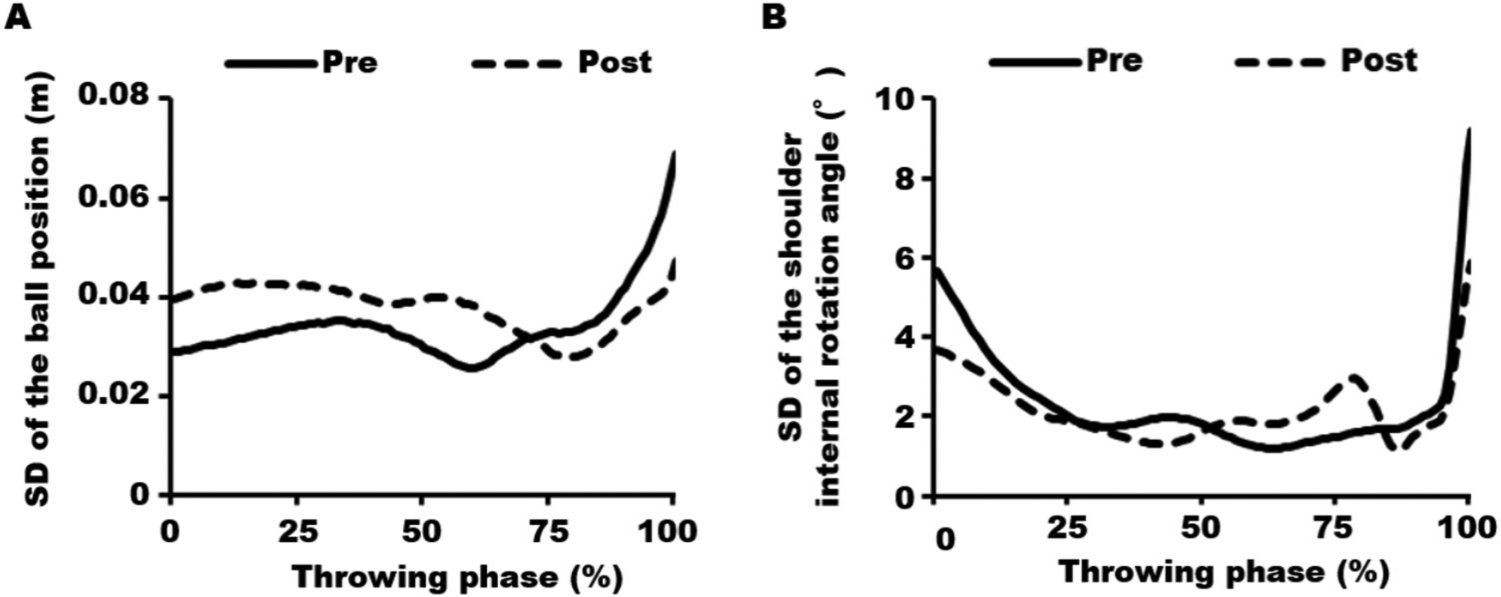
Variability in the ball position and shoulder internal rotation angle. **(A)** The variability (standard deviation) of the three-dimensional ball positions before the intervention (solid line) increased rapidly just before ball release (100% of the throwing phase). After the intervention (dashed line), the variability in ball position at ball release decreased. **(B)** The variability of the shoulder internal rotation angle before the intervention (solid line) increased rapidly just before ball release (100% of the throwing phase). After the intervention (dashed line), the variability in the shoulder internal rotation angle at ball release decreased.

Table 2 details the results for the ball release parameters. Supplementary Figure S1 illustrates the three-dimensional trajectories and release points of the ball. The figure demonstrates that, following the intervention, the variability of ball release points and the release angle immediately after release were both reduced. Specifically, variability in the antero-posterior and vertical components of the release position decreased by 35.6% and 30.4%, respectively. However, variability in the horizontal release position showed no noticeable change. The time course changes in the variability of the three-dimensional ball position are depicted in Figure 2A. The variability in the ball position was abruptly higher toward the ball release point before the intervention but was lower after the intervention.

### 3.4 Upper limb kinematics at ball release

Table 2 summarized the results for upper limb kinematic data at ball release. The time course changes in the variability of the shoulder joint angle are depicted in Figure 2B. Before the intervention, the greatest variability in joint angles at ball release was observed in shoulder internal rotation, which decreased from 9.2° to 5.9° following the intervention. In contrast, variability in wrist dorsiflexion exhibited a slight increase, rising from 4.5° to 6.1°. Variability in other joint angles—shoulder abduction, horizontal abduction, and elbow flexion—remained relatively low before and after the intervention.

## 4 Discussion

This case report examined the effects of task-oriented throwing training on a baseball player with symptoms of throwing yips. Our results showed that the task-oriented throwing training promoted improvements in the ball arrival position accompanied by a reduction in the variability in the ball release position, joint angle at ball release, and ball release angle. These results have considerable clinical significance as they are the first to show that task-oriented training may be effective in treating yips symptoms occurring among athletes.

### 4.1 Yips symptoms before the intervention

The yips have traditionally been classified as either task-specific dystonia or choking; however, these two conditions are not mutually exclusive and are now understood to lie along a continuum (19, 20). Herein, the participant's symptoms initially emerged after a psychologically stressful incident. Despite this, he later displayed ongoing motor performance decline and pronounced arm stiffness even in situations devoid of psychological pressure. This case highlights the intricate interaction between psychological and neurological factors in the development and persistence of symptoms, aligning with the contemporary perspective that the yips exist on a spectrum that incorporates elements of both domains.

The yips symptoms in this case changed markedly depending on the throwing conditions. Similarly, reports on golfers' yips have shown that short-distance putting of approximately 3 to 4 ft. is more likely to reproduce the symptoms than would longer distance putting (1). Moreover, evidence suggests that the intensity of symptoms in baseball players with throwing yips varies depending on the throwing distance and throwing strength (5, 11). These previous studies support the observation that yips symptoms can vary based on throwing conditions, as seen in this case. Herein, we leveraged the task- and context-dependent nature of these symptom fluctuations to design a task-oriented throwing training program that effectively alleviated the athlete's symptoms. This finding underscores the importance of carefully assessing how symptoms change with different throwing tasks or environments to develop individualized training approaches tailored to each athlete's specific presentation.

Prior to the intervention, the player exhibited impaired throwing accuracy, possibly due to increased ball release angle variability. Normally, the shoulder joint rapidly rotates internally from a maximum external rotation position until ball release (21), suggesting that an early ball release timing results in insufficient shoulder internal rotation, whereas a delayed ball release timing promotes greater shoulder internal rotation. Therefore, the variability in the shoulder internal rotation angle at ball release may be attributed to the variability in the ball release timing. Hore et al., who investigated the relationship between the ball arrival position and upper limb kinematics in healthy participants (22), showed that the variability in ball release timing and the resulting kinematic variability at ball release have an important impact on the ball arrival position, supporting our findings. Therefore, the extremely large variability in the throwing motion observed in the current case prior to intervention may be interpreted as a condition wherein the normal range of motor variability is exaggerated.

Few previous studies have investigated the variability in movements exhibited by athletes with the yips. Among golfers' yips, substantial variability in forearm acceleration during the putting motion and face orientation at impact has been observed (23, 24). The present results are valuable for supporting the findings of these studies and for indicating that impaired movement variability may be associated with diminished motor skills among athletes with the yips in sports other than golf.

### 4.2 The effect of task-oriented throwing training on yips symptoms

After the intervention, the frequency at which the subjective yips symptoms were reproduced during throwing was reduced by around one-third. Accompanying these changes, the variability in the ball release position and ball release angle was also reduced, thereby improving throwing accuracy. Furthermore, the variability in the internal rotation angle of the shoulder joint at ball release was reduced after the intervention. As mentioned earlier, the ball release parameters and variability in kinematics at ball release are likely dependent on the ball release timing. Therefore, graded task-oriented throwing training tailored to individual players' symptoms may improve ball release timing and the associated ball release parameters and upper limb kinematic variability at ball release, thereby improving throwing accuracy. Few studies have documented the effectiveness of exercise therapy in alleviating yips symptoms. Although Katz et al. examined the effect of therapeutic interventions on lower limb focal task-specific dystonia in athletes (9), the treatment approach included various methods such as botulinum toxin injections, medication, and orthotics alongside exercise therapy. Therefore, the extent to which exercise therapy was effective in treating symptoms of the yips remains unclear. Recently, Giorgi et al. (25) reported a case demonstrating the effectiveness of an integrated rehabilitation program—combining postural and neuromuscular rehabilitation—for lower limb dystonia in a football player. After 7 months, the athlete showed symptom reduction and returned to near presymptom performance levels. In their report, no concomitant medication or botulinum toxin injections were used, which is consistent with our results. Taken together, available evidence suggests that rehabilitative approach may have the potential to reduce the yips symptoms not only in the lower limb but also in the upper limb. Future large-scale studies, including athletes with the yips in other sports, will be necessary to confirm our findings.

### 4.3 Limitations

This study has several notable limitations. Although it focused on changes in kinematic characteristics, it did not evaluate psychological variables or potential shifts in autonomic nervous system activity—such as heart rate variability—that may accompany psychological changes. Investigating the interaction between psychological factors and motor symptoms in the yips represents a promising direction for future research. Although the intervention led to a reduction in symptoms for the current participant, it did not fully eliminate them. This may be partially attributed to the increased variability in wrist joint angles at ball release following the intervention. As such, future treatment approaches should place greater emphasis on improving distal joint control, particularly wrist mechanics, to further reduce symptoms. Furthermore, the study did not examine the duration of symptom relief after the intervention—a key limitation that should be considered when interpreting the results.

## 5 Conclusions

This case report examined the effects of a graded task-oriented throwing training on a baseball player who presented with throwing yips. Our findings showed that kinematic variability at ball release decreased after the intervention, thereby improving throwing accuracy. Medication and injections are rarely available as treatment options for symptoms of the yips among athletes due to side effects and doping issues. Therefore, the findings of the present study, which showed that this training with its low risk of such problems may represent a potentially effective treatment strategy for the yips, could have important clinical implications. Further studies involving larger sample sizes are needed in the future.

## Data Availability

Data supporting the findings of this study are available from the authors upon reasonable request.

## References

[1] Smith AM Malo SA Laskowski ER Sabick M Cooney WP Finnie SB A multidisciplinary study of the ‘yips’ phenomenon in golf: an exploratory analysis. Sports Med. (2000) 30:423–37. 10.2165/00007256-200030060-00004 11132124

[2] Bawden M Maynard I. Towards an understanding of the personal experience of the ‘yips’ in cricketers. J Sports Sci. (2001) 19:937–53. 10.1080/026404101317108444 11820688

[3] Aoyama T Ae K Taguchi T Kawamori Y Sasaki D Kawamura T Spatiotemporal patterns of throwing muscle synergies in yips-affected baseball players. Sci Rep. (2024) 14:2649. 10.1038/s41598-024-52332-9 38302478 PMC10834996

[4] Stinear CM Coxon JP Fleming MK Lim VK Prapavessis H Byblow WD. The yips in golf: multimodal evidence for two subtypes. Med Sci Sports Exerc. (2006) 38:1980–9. 10.1249/01.mss.0000233792.93540.10 17095933

[5] Aoyama T Ae K Souma H Miyata K Kajita K Nara T A feasibility study of the incidence and symptoms of the throwing yips in college baseball players (in Japanese). Japanese J Phys Fit Sports Med. (2021) 70:91–100. 10.7600/jspfsm.70.91

[6] van Wensen E Nijenhuis B Zwerver J Bogaard P-J. An EMDR-based intervention for the ‘Golfers’ Yips’: a case series. Sports Psy. (2024):10.1024/2674-0052/a000083. 10.1024/2674-0052/a000083

[7] Bell RJ Skinner CH Fisher LA. Decreasing putting yips in accomplished golfers via solution-focused guided imagery: a single-subject research design. J Appl Sport Psychol. (2009) 21:1–14. 10.1080/10413200802443776

[8] Bell RJ Skinner CH Halbrook MK. Solution-focused guided imagery as an intervention for golfers with the yips. J Imagery Res Sport Phys Act. (2011) 6:1–16. 10.2202/1932-0191.1059

[9] Katz M Byl NN San Luciano M Ostrem JL. Focal task-specific lower extremity dystonia associated with intense repetitive exercise: a case series. Parkinsonism Relat Disord. (2013) 19:1033–8. 10.1016/j.parkreldis.2013.07.013 23932354

[10] Shukla AW Hu W Jabarkheel Z Shah S Legacy J Firth KN Globus pallidum DBS for task-specific dystonia in a professional golfer. Tremor Other Hyperkinet Mov. (2018) 8:487. 10.7916/D83X9Q9D PMC6214811 30402336

[11] Aoyama T Ae K Souma H Miyata K Kajita K Kawamura T Difference in personality traits and symptom intensity according to the trigger-based classification of throwing yips in baseball players. Front Sports Act Living. (2021) 3:652792. 10.3389/fspor.2021.652792 34514382 PMC8424038

[12] Rensink M Schuurmans M Lindeman E Hafsteinsdottir T. Task-oriented training in rehabilitation after stroke: systematic review. J Adv Nurs. (2009) 65:737–54. 10.1111/j.1365-2648.2008.04925.x 19228241

[13] Salvalaggio S Gianola S Ando M Cacciante L Castellini G Lando A Predictive factors and dose-response effect of rehabilitation for upper limb induced recovery after stroke: systematic review with proportional meta-analyses. Physiotherapy. (2024) 125:101417. 10.1016/j.physio.2024.101417 39395360

[14] Shimonaka J Nakazato K Gondo K Takayama M. Revised NEO-Personality Inventory (NEO-PI-R) and NEO Five-Factor Inventory (NEO-FFI) Manual for the Japanese Version. Tokyo: Tokyo Shinri (1999).

[15] Hedano T Hukuhara M Iwawaki S Soga S Spielberger CD. State Trait Anxiety Inventory-Form JYZ Test Manual. (Japanese Version of STAI). Tokyo: Jitsumukyoiku-syuppan (2000).

[16] van der Kruk E Reijne MM. Accuracy of human motion capture systems for sport applications; state-of-the-art review. Eur J Sport Sci. (2018) 18:806–19. 10.1080/17461391.2018.1463397 29741985

[17] Eldemir S Guclu-Gunduz A Eldemir K Saygili F Yilmaz R Akbostanci MC. The effect of task-oriented circuit training-based telerehabilitation on upper extremity motor functions in patients with Parkinson’s disease: a randomized controlled trial. Parkinsonism Relat Disord. (2023) 109:105334. 10.1016/j.parkreldis.2023.105334 36917914

[18] Toovey R Bernie C Harvey AR McGinley JL Spittle AJ. Task-specific gross motor skills training for ambulant school-aged children with cerebral palsy: a systematic review. BMJ Paediatrics Open. (2017) 1:e000078. 10.1136/bmjpo-2017-000078 29637118 PMC5862184

[19] Smith AM Adler CH Crews D Wharen RE Laskowski ER Barnes K The ‘yips’ in golf: a continuum between a focal dystonia and choking. Sports Med. (2003) 33:13–31. 10.2165/00007256-200333010-00002 12477375

[20] Revankar GS Kajiyama Y Gon Y Ogasawara I Hattori N Nakano T Perception of yips among professional Japanese golfers: perspectives from a network modelled approach. Sci Rep. (2021) 11:20128. 10.1038/s41598-021-99128-9 34635697 PMC8505642

[21] Dillman CJ Fleisig GS Andrews JR. Biomechanics of pitching with emphasis upon shoulder kinematics. J Orthop Sports Phys Ther. (1993) 18:402–8. 10.2519/jospt.1993.18.2.402 8364594

[22] Hore J Watts S Tweed D. Errors in the control of joint rotations associated with inaccuracies in overarm throws. J Neurophysiol. (1996) 75:1013–25. 10.1152/jn.1996.75.3.1013 8867114

[23] Marquardt C. The vicious circle involved in the development of the yips. Int J Sports Sci Coach. (2009) 4:67–88. 10.1260/174795409789577506

[24] Adler CH Temkit M Crews D McDaniel T Tucker J Hentz JG The yips: methods to identify golfers with a dystonic Etiology/Golfer’s cramp. Med Sci Sports Exerc. (2018) 50:2226–30. 10.1249/MSS.0000000000001687 29889820

[25] Giorgi V Apostolo G Bertele L. Treating dystonia in a soccer player through an integrated rehabilitative approach: a case report. J Sport Rehabil. (2024) 33:365–75. 10.1123/jsr.2023-0100 38702050

