# Supplementary material for: Case Report: Effect of a graded task-oriented throwing training on throwing accuracy and kinematic variability in a baseball player with the yips
Source: Front Sports Act Living. 2025 Aug 29;7:1636650. doi: 10.3389/fspor.2025.1636650 (PMC12426106; doi:10.3389/fspor.2025.1636650)
Supplement: Supplementary file 1 [file Datasheet1.pdf]

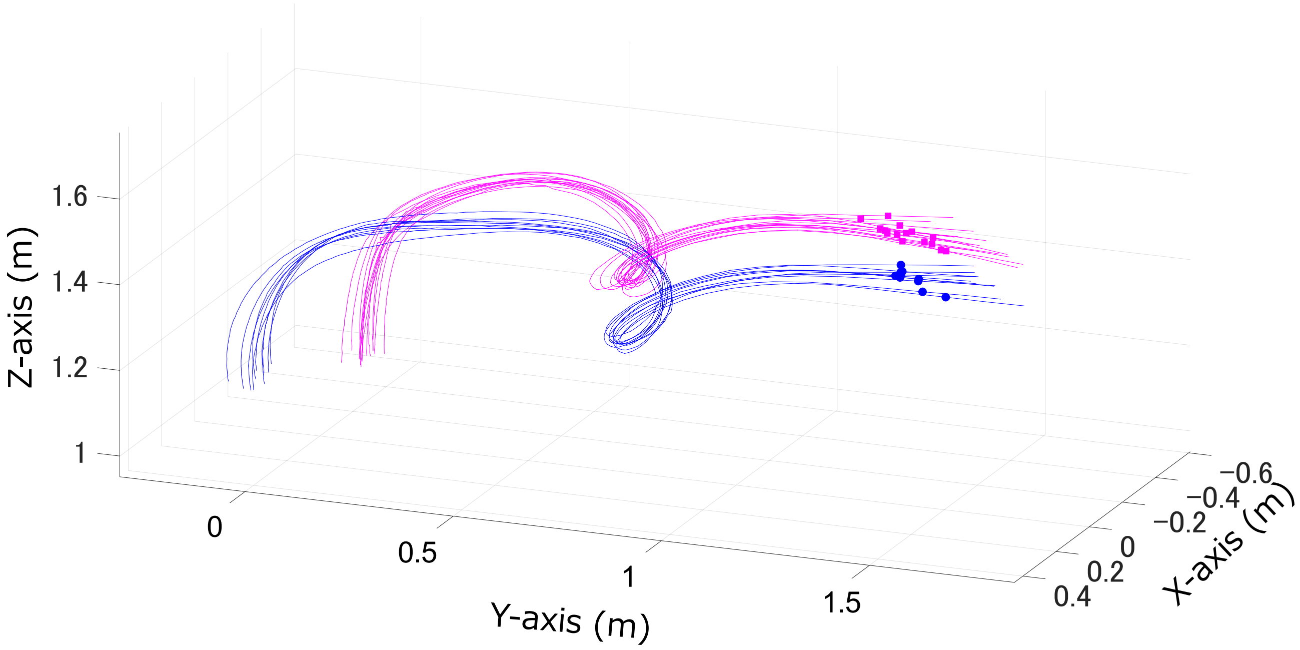

Supplement figure 1. Three-dimensional ball trajectories and release points before and after the intervention

Pink (before) and blue (after) lines represent the ball trajectories during throwing, spanning from 0% to just after release. Pink squares and blue circles indicate the release points for each individual trial before and after the intervention. Axes represent spatial coordinates in the X, Y, and Z directions.
